# Fast Underwater Optical Beacon Finding and High Accuracy Visual Ranging Method Based on Deep Learning

**DOI:** 10.3390/s22207940

**Published:** 2022-10-18

**Authors:** Bo Zhang, Ping Zhong, Fu Yang, Tianhua Zhou, Lingfei Shen

**Affiliations:** 1 College of Science, Donghua University, Shanghai 201620, China; 2 Key Laboratory of Space Laser Communication and Detection Technology, Shanghai Institute of Optics and Fine Mechanics, Chinses Academy of Sciences, Shanghai 201800, China

**Keywords:** autonomous underwater vehicles, target detection, monocular vision, deep learning

## Abstract

Visual recognition and localization of underwater optical beacons is an important step in autonomous underwater vehicle (AUV) docking. The main issues that restrict the use of underwater monocular vision range are the attenuation of light in water, the mirror image between the water surface and the light source, and the small size of the optical beacon. In this study, a fast monocular camera localization method for small 4-light beacons is proposed. A YOLO V5 (You Only Look Once) model with coordinated attention (CA) mechanisms is constructed. Compared with the original model and the model with convolutional block attention mechanisms (CBAM), and our model improves the prediction accuracy to 96.1% and the recall to 95.1%. A sub-pixel light source centroid localization method combining super-resolution generative adversarial networks (SRGAN) image enhancement and Zernike moments is proposed. The detection range of small optical beacons is increased from 7 m to 10 m. In the laboratory self-made pool and anechoic pool experiments, the average relative distance error of our method is 1.04 percent, and the average detection speed is 0.088 s (11.36 FPS). This study offers a solution for the long-distance fast and accurate positioning of underwater small optical beacons due to their fast recognition, accurate ranging, and wide detection range characteristics.

## 1. Introduction

Remotely Operated Vehicles (ROV) and Autonomous Underwater Vehicles (AUV) are the two main forms of Unmanned Underwater Vehicles (UUV), which are crucial tools for people exploring the deep sea [1,2,3,4]. Because of the restriction on cable length, ROVs can only operate within a certain range and are dependent on the control platform and operator, which limits their flexibility and prevents them from meeting the requirements for military operations regarding concealment. While AUV overcomes the shortcomings of ROV and has better flexibility and concealment because they do not have the limitations of cables and operating platforms, AUV technology is gradually becoming the focus of national marine research [5,6]. However, AUV is limited by the electromagnetic shielding of the water column, and existing communication technologies on land are difficult to adapt to the communication needs of the entire water column [7]. In order to exchange information and charge its batteries, the AUV must return frequently to dock with the supply platform. Typically, the current AUV return solution uses an inertial navigation system (INS) for positioning over long distances, an ultra-short base-line positioning system at a distance of about one kilometer from the docking platform, and a multi-sensor fusion navigation method to approach the docking interface [8,9,10]. When the AUVs are more than 10 m away from the docking interface, the aforementioned technique struggles to meet the demands of precise positioning and docking [11]. Installing identification markers, such as light sources on the AUV and the docking interface, is the current standard procedure for increasing docking accuracy. Additionally, machine vision technology is used in the underwater environment to gather the necessary direction and distance information by sensing the characteristics of the identification markers [12,13,14].

Using traditional machine vision and image processing methods, Lijia Zhong et al. proposed a binocular vision localization method for AUV docking, using an adaptive weighted OTSU threshold segmentation method to accurately extract foreground targets. The method achieves an average position error of 5 cm and an average relative error of 2% within a range of 3.6 m [15]. This technique uses binocular vision to increase detection accuracy. However, doing so increases the size of the equipment, which raises costs, reduces the method’s potential application areas, and adds pressure on the deep water.

In recent years, the underwater positioning method of AUVs based on deep learning has also been widely developed. Shuang Liu et al., based on the idea of MobileNet, built a Docking Neural Network (DoNN) with a convolutional neural network, realized the target extraction of the interface through a monocular camera, and combined the RPnP algorithm to convert the 8-LED with a size of 2408 mm. The detection range of the docking interface is increased to 6.5 m, and under the condition of strong noise, the average detection error of the docking distance is only 9.432 mm, and the average detection error of the angle is 2.353 degrees [16]. Ranzhen Ren et al. used the combination of YOLO v3 and the P4P algorithm to locate an optical beacon with a length of 28 cm at a long distance of 3 m to 15 m and used Aruco markers for positioning within a range of 3 m, realizing the visual docking of the combination of far and near [17].

The underwater ranging algorithm combined with deep learning has been proven to have the advantages of better real-time performance and higher detection accuracy [17,18,19]. However, the following problems need to be solved:

1. The water pressure increases as the AUV’s navigation depth and volume increase. Therefore, it is necessary to reduce the size of the AUV and improve the ranging accuracy of the monocular camera. This poses a high demand for long-distance identification of small optical beacons, and the existing target detection algorithms are difficult to meet;
2. With the increase in AUV working distance and the decrease in the size of the optical beacons, the light source characteristics of the optical beacon can only occupy a few pixel sizes, which makes it very difficult to locate the centroid pixel coordinates of the light source;
3. The traditional Perspective-n-point (PnP) algorithm for optical beacon attitude calculation has low accuracy for long-distance target pose.

In order to solve the above problems, the main contribution and core innovation of this paper are as follows:

- In order to solve the problem of long-distance target recognition of small optical beacons, YOLO V5 is used as the backbone network [20], and the Coordinate Attention (CA) and Convolution Block Attention Mechanisms (CBAM) are added for comparison [21,22], and training is performed on a self-made underwater optical beacon data set. It is proved that the YOLO v5 model, by adding CA has good detection accuracy for small optical beacons when the network depth is relatively shallow and solves the problem of difficulty in extracting small optical beacons at 10 m underwater;
- In order to solve the problem of difficulty in obtaining pixel coordinates due to the small number of pixels occupied by the feature points of small optical beacons, a Super-Resolution Generative Adversarial Network (SRGAN) was introduced into the detection process [23]. Then, the sub-pixel coordinates of the light source centroid are obtained through adaptive threshold segmentation (OTSU) and the sub-pixel centroid extraction algorithm based on Zernike moments [24,25]. It is proved that the combination of super-resolution and sub-pixel has a good effect on the localization of the pixel coordinates of the target light source in the case of 4-time upscaling reconstruction of the image;
- In order to solve the problem of inaccurate calculation of the pose of small optical beacons, a simple and robust perspective-n-point algorithm (SRPnP) is used as the pose solution method, and it is compared with the non-iterative O(n) solution of the PnP problem (OPnP) and one of the best iterative methods, which is globally convergent in the ordinary case(LHM) [26,27,28].

Our method adds an attention mechanism to the classical neural network model YOLO V5, migrates the detection algorithm applied to land to the water environment, and improves the precision and recall of the model. The super-resolution technology is innovatively used as an image enhancement method for small optical beacons and is combined with the sub-pixel centroid positioning method to improve the range and accuracy of light source centroid positioning. Through the experimental verification, the distance calculation error of the proposed method for four-light source small optical beacons with a size of 88 mm × 88 mm is 1.04%, and the average detection speed is 0.088 s (11.36 FPS). Our method is superior to existing methods in detection accuracy, detection speed, and detection range and provides a feasible and effective method for monocular visual ranging of underwater optical beacons.

## 2. Experimental Equipment and Testing Devices

**Figure 1:**
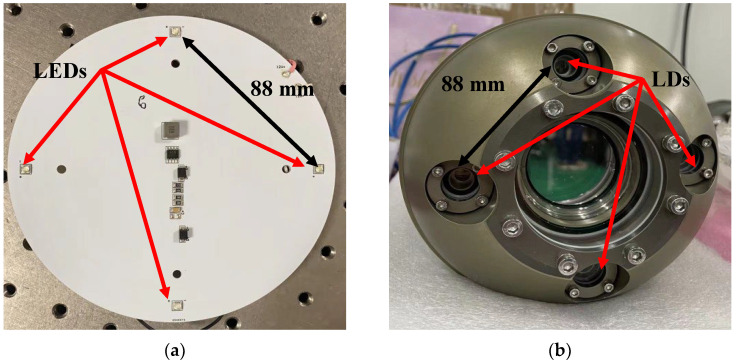
Optical beacons: (**a**) A cross-light beacon composed of three 520 nm LEDs and one 450 nm LED; (**b**) an optical communication probe composed of four 520 nm laser diodes.

For the experiments, underwater optical beacon images between 1 and 10 m deep were gathered. There are two different categories of underwater optical beacons (Figure 1): cross beacons made of four light-emitting diodes (LEDs) and cross optical communication probes made of four laser diodes (LDs). The wavelengths of the two optical beacon light sources are both 520 nm and 450 nm because these two wavelengths of light have the least propagation attenuation in seawater [29]. The distance between two adjacent light sources is 88 mm, and the diagonal light source spacing is 125 mm.

**Figure 2:**
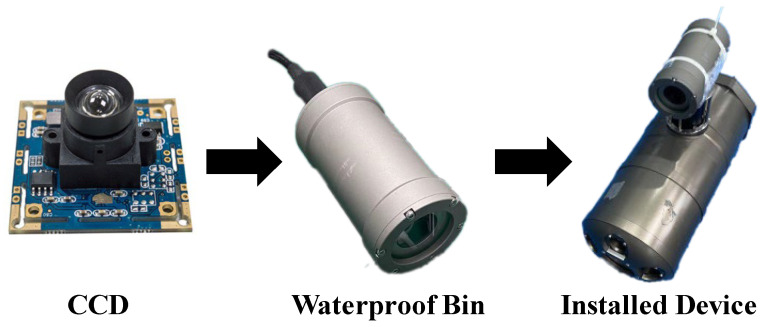
Underwater image acquisition device combined with optical communication probe.

A Sony 322 camera that took pictures and was mounted in a waterproof compartment, along with an optical communication probe, was used to conduct underwater experiments in the range of up to 10 m in an anechoic pool, as shown in Figure 2. A Cannon D60 was used in a temporary lab pool to capture the high-resolution optical beacon images at a range of 3 m, because a super-resolution dataset was needed.

**Figure 3:**
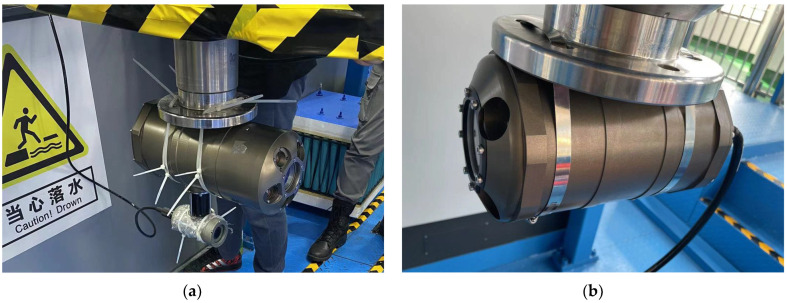
Device installation diagram: (**a**) The detection device is fixed to the lift bar and drops to 3 m underwater; (**b**) The device to be tested is fixed to a lift rod on the other side and lowered to 3 m underwater.

In the underwater ranging experiment, the detection probe and the probe to be measured are fixed on two high-precision lifting and slewing devices on two precision test platforms. The lifting rod can move in three dimensions and have its end rotate 360 degrees at the same time (Figure 3). The accuracy of the three-dimensional movement of the translation stage is 1 mm, and the rotation error is 0.1 degree. In the experiment, two rotating lifting rods will control the device to descend 3 m below the water surface and keep the depth constant. The devices only change the rotation angle and the relative position of the same plane.

**Figure 4:**
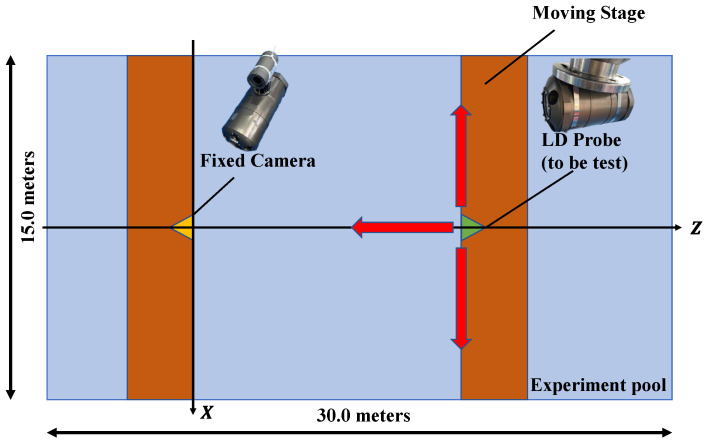
The condition of the experiment (top view).

The devices only change the rotation angle and the relative position of the same plane. Figure 4 is a top view of the experimental conditions for image acquisition in this paper. The experiment was carried out in an anechoic tank with a width of 15 m and a length of 30 m. Ensure that the device equipped with the camera is stationary, the device to be tested is fixed on the mobile platform and moves along the *z*-axis, and only fine-tuning in the *x*-axis direction makes the optical beacon feature appear in the CCD field of view. Initially, the two platforms were separated by 10 m, and images were collected every 1 m.

## 3. Underwater Optical Beacon Target Detection and Light Source Centroid Location Method

**Figure 5:**
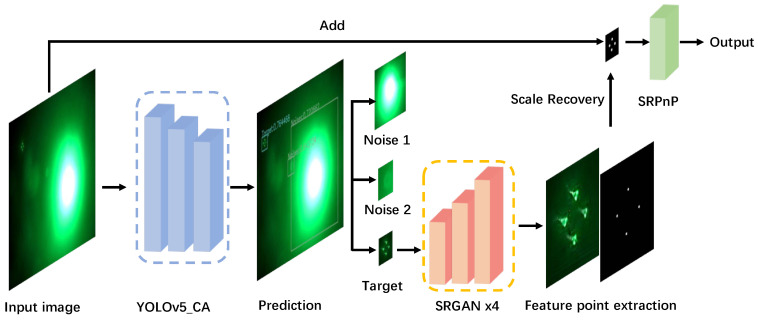
Flow chart of visual positioning algorithm.

Figure 5 depicts our method’s overall workflow: The algorithm’s input is the underwater optical beacon image captured by the calibrated CCD. The target is detected using YOLO V5 CA, and the target image is then fed into SRGAN for 4× super-resolution enhancement. After obtaining the subpixel quality coordinates of the feature light source using the adaptive OTSU threshold segmentation and Zernike sub-pixel edge detection, scale recovery is used to obtain the image coordinates of the feature points within the original image size. The SRPnP algorithm is then used to determine the target’s pose and distance.

### 3.1. Underwater Target Detection Method Based on YOLO V5

It is very challenging to locate and extract underwater targets because of the complexity of the underwater environment, including the light deflection caused by water flow, the reflection formed by the light water surface and the waterproof chamber, and the environmental disturbances. YOLO V5 (You Only Look Once), as a fast and accurate target recognition neural network, is used in the target detection stage.

YOLO V5 follows the main structure and regression classification method of YOLO V4 [30]. However, it also includes the most recent techniques, including CIoUloss, adaptive anchor box calculation, and adaptive image scaling, to help the network converge more quickly and detect targets with greater accuracy during training. And the extraction of overlapping targets is also better than that of YOLO V4. The most important thing is that it reduces the size of the model to 1/4 of the original model, which meets the real-time detection requirements of underwater equipment in terms of detection speed. The loss function used by YOLO V5 for training is

(1) $$\begin{array}{ll} Loss= & CIoUloss\\ & +\sum_{i=0}^{S^{{}^{2}}}\sum_{j=0}^{B_{n}}I_{ij}^{obj}\left[C_{i}\log\left(C_{i}\right)+\left(1-C_{i}\right)\log\left(1-C_{i}\right)\right]\\ & +\sum_{i=0}^{S^{{}^{2}}}\sum_{j=0}^{B_{n}}I_{ij}^{noobj}\left[C_{i}\log\left(C_{i}\right)+\left(1-C_{i}\right)\log\left(1-C_{i}\right)\right]\\ & +\sum_{i=0}^{S^{2}}\sum_{j=0}^{B_{n}}I_{ij}^{obj}\sum_{c\in classes}\left[p^{j}{}_{i}\left(c\right)\log\left(p^{j}{}_{i}\left(c\right)\right)+\left(1-p^{j}{}_{i}\left(c\right)\right)\log\left(1-p^{j}{}_{i}\left(c\right)\right)\right] \end{array}$$

In Formula (1), S is the number of grid cells into which the input image is divided, $B_{n}$ is the number of anchor boxes, CIoUloss is the loss of the bounding box. The second and third terms are confidence loss, which means the confidence of the bounding box containing the object $I_{ij}^{obj}$ and the confidence of the bounding box without object $I_{ij}^{noobj}$. The fourth term is the cross-entropy loss. When the jth anchor frame of the ith grid is responsible for the prediction of a real target, the bounding box generated by this anchor frame is only involved in the calculation of the classification loss function. Assuming that A is the prediction box and B is the real box, let C be the minimum convex closed box containing A and B, then the intersection over union (IoU) of the real box and the prediction box and the loss function CIoUloss are calculated as follows:

(2) $$IoU=\frac{\left|A\cap B\right|}{\left|A\cup B\right|},$$

(3) $$CIoU=IoU-\frac{{\mathrm{Distance}\text{\_}\mathrm{Center}}^{2}}{{\mathrm{Distance}\text{\_}\mathrm{Corner}}^{2}}-\frac{v^{2}}{\left(1-IOU\right)+v},$$

where

$$v=\frac{4}{\pi^{2}}{\left(\arctan\frac{\hat{w}}{\hat{h}}-\arctan\frac{w}{h}\right)}^{2},$$

CIoUloss=1−CIoU.

In Formula (3), Distance_Center is the Euclidean distance between the center points of A box and B box, Distance_Corner is the diagonal length of the box, and v is a parameter to measure the consistency of the aspect ratio between the predicted box and the real box.

### 3.2. YOLO V5 with Attention Modules

Fast object detection and classification are advantages of the YOLO V5n model, but this is due to a reduction in the number and width of network layers, which also has the drawback of lowering detection accuracy. The application of attention mechanisms in machine vision enables the network model to ignore irrelevant information and focus on important information. The attention mechanisms can be divided into spatial domains, channel domains, mixed domains, etc. Therefore, this paper adds the CBAM attention mechanism and the coordinate attention mechanism to the YOLO V5n model, trains it on the self-made underwater light beacon dataset, and compares the validation set loss, recall rate, and mean average precision (Map). It is proven that the CA attention mechanism has a good improvement over the lightweight YOLO V5 model and realizes the long-distance accurate identification and detection of small optical beacons in the water environment.

**Figure 6:**
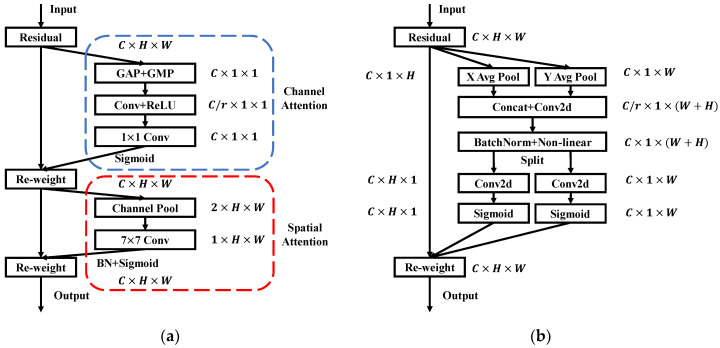
Diagram of attentional mechanism: (**a**) Convolutional block attention mechanism; (**b**) Coordinate attention mechanism.

As seen in Figure 6a, the Convolutional Block Attention Module (CBAM) is a module that combines spatial and channel attention mechanisms. The input feature image first passes through a residual module, and then performs global maximum pooling (GMP) and global average pooling (GAP) to obtain two one-dimensional feature vectors, which are then subjected to convolution, ReLU activation, and 1 × 1 Convolution, weighted with input features after normalization using Batch Normal (BN) and Sigmoid, to obtain feature maps for channel attention. The channel feature map performs GAP and GMP on all channels at each pixel position to obtain two feature maps, and after 7 × 7 convolutions of the two feature maps, normalization is performed to obtain the attention feature that combines channel and space.

The channel feature map contains the local area information in the original image after several convolutions. The global feature information of the original image cannot be obtained because only the local information is taken into account when the maximum and average values of the multi-channels at each position are used as weighting operations. This issue is effectively resolved by the coordinate attention mechanism, as seen in Figure 6b: the feature map passing through the residual module uses the pooling kernel of summation along the horizontal coordinate (H, 1) and vertical coordinate (1, W) pairs respectively. Each channel is encoded to obtain the features of two spatial orientations. This method allows the network to obtain the feature information in one spatial direction and save the other spatial position information, which helps the network to locate the target of interest more accurately.

**Figure 7:**
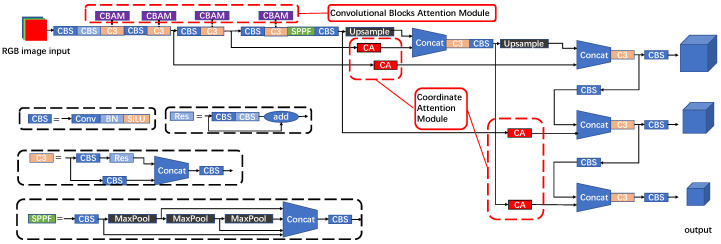
YOLO v5 structure diagram with attention modules.

A schematic representation of the YOLO V5 structure with attention modules is shown in Figure 7. Two structures were created to fit the CBAM and CA characteristics. Four C3 convolution modules with different depths in the backbone network are replaced by CBAM with the same input and output to address the issue that multiple convolutions cause CBAM to lose local information. This allows CBAM to obtain weights for channel and spatial feature maps at various depths. Due to the lightweight of the CA modules, they are added to the positions of the 4-feature map fusion, so that the model can better obtain the weight of different feature maps.

**Figure 8:**
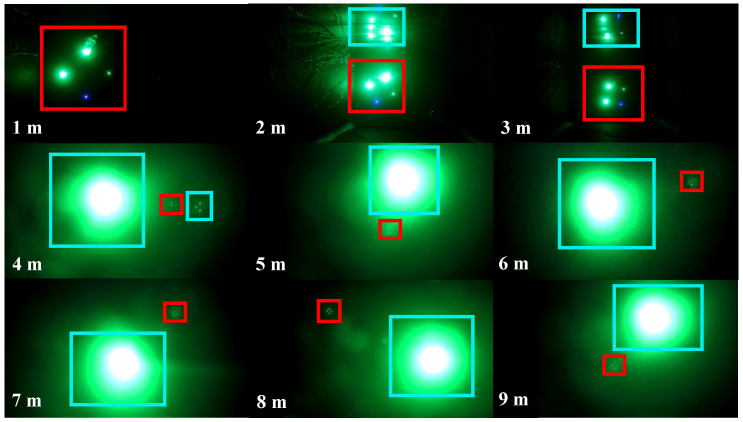
Pictures of LED and LD optical beacons collected within 1–10 m.

In Figure 8, the target is the object picked by the red boxes, and the mirror image of the target and the water’s surface, as well as the laser point for optical communication, are selected by the blue boxes. Because the objects chosen by the blue box during image acquisition are noise and will interfere with the extraction of the target, the labels are divided into target and noise. This makes it necessary to distinguish between noise and target features using neural network training. 7606 images of LED and LD optical beacons with various attitudes were collected in the underwater environment ranging from 1 m to 10 m, of which 5895 were training set data, and 1711 were test set data. Three models of YOLO V5n, YOLO V5_CBAM, and YOLO V5_CA are trained separately on RTX 3090 with a 128-batch size and 500 epochs. To prevent overfitting of the model, an early stop mechanism was introduced during the training where YOLO V5n stopped at 496, and YOLO V5_CA stopped at 492.

**Figure 9:**
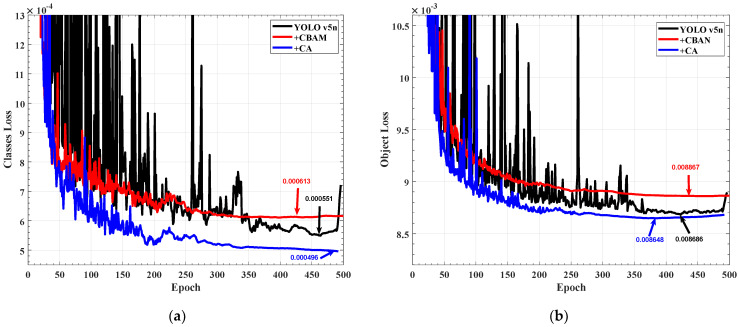
Verification set loss: (**a**) Verification set classification loss; (**b**) Verification set object loss.

An essential parameter to gauge the discrepancy between the predicted value and the true value is the loss of the validation set. Within 500 training rounds, all three of the models in Figure 8 had reached convergence. In Figure 9a, the class loss of the model with the CA module is $4.96\times 10^{-4}$, the initial model is $5.51\times 10^{-4}$, and the model with CBAM is $6.13\times 10^{-4}$. In Figure 9b, the object loss of the model with the CA module is $8.65\times 10^{-3}$, the initial model is $8.69\times 10^{-3}$, and the model with CBAM is $8.87\times 10^{-3}$. According to the above data, the model with the CA module has improved compared with the initial model in both target detection and classification, but the model with CBAM has degenerated.

**Figure 10:**
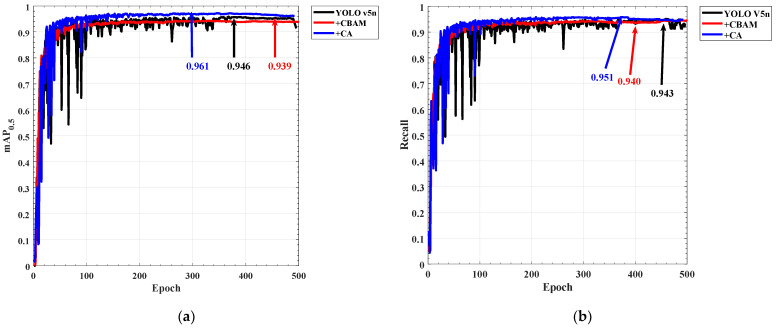
Comparison of network training results: (**a**) Mean of Average Precision; (**b**) Recall.

The mean of average precision (mAP), which is a crucial measurement of the classification accuracy of the model, refers to the average prediction accuracy of each type of target and then the average value of the entire dataset. The recall is a crucial indicator to determine whether the target object has been examined. As shown in Figure 10a, the maximum mAP of the network with the CA module is 96.1%, the maximum mAP of the network with CBAM is 93.9%, and the maximum mAP of the original YOLO V5n model is 94.6%. It can be seen that the CA module improves the classification accuracy by 1.5%, while the CBAM reduces the classification accuracy by 0.7%. The reason why the CBAM module causes network degradation is that the long-distance optical signal object and the mirror target in the training image have high consistency, the target scale is small, and the global features cannot effectively separate the noise and the target. As shown in Figure 10b, the recall rate of the target by the CA module is also improved by 0.8%. In the detection of distant targets, the network with CA has a good effect on recognition and classification. Therefore, in the subsequent experiments to verify the model detection effect, the YOLO V5_CA and YOLO V5n models are used for comparison.

**Figure 11:**
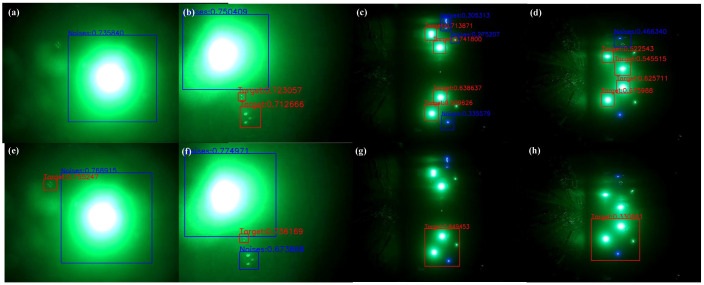
Target detection result: (**a**–**d**) are YOLO V5n detection results; (**e**–**h**) are YOLO V5_CA detection results.

Figure 11 is a comparison chart of the detection results of YOLO V5n and YOLO V5_CA. It can be seen that when the characteristics of the target object are not obvious, there will be a problem of missed detection in the detection of the original model (Figure 11a). When objects and noises with very similar characteristics appear at the same time (Figure 11b), the original model will have the problem of misidentifying the noise as a target. When detecting close-range LED targets (Figure 11c,d), the original model will fail to detect them. The model with the CA module has a good classification effect on the target and noise, and the detection accuracy is higher than the original model.

**Figure 12:**
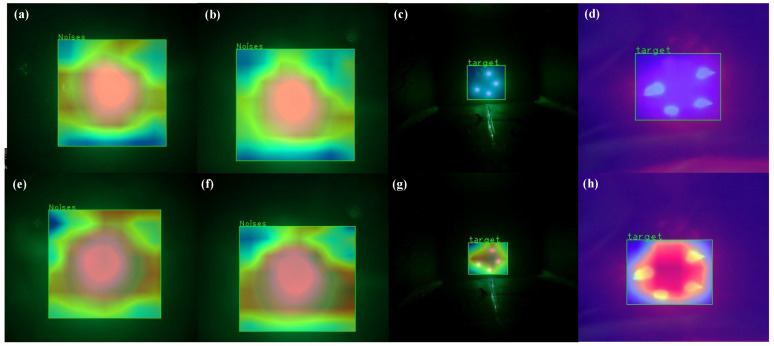
Heat maps: (**a**–**d**) are heat maps of YOLO V5n; (**e**–**h**) are heat maps of YOLO V5_CA.

We used Grad-CAM as a visualization tool for our network [31]. The region that the network is most interested in is the one with the redder in Figure 12. It is clear that YOLO V5 with the CA module outperforms the original model in beacon extraction and localization for both noise and light. As a result, in the underwater target recognition and extraction stage, the recognizer is YOLO v5 with the CA module.

### 3.3. SRGAN and Zernike Moments-Based Sub-Pixel Optical Center Positioning Method

After correctly identifying and extracting the target, the size of the light source feature points is only a few to a dozen pixels because the scale of the target at about 10 m is extremely small. Conventional image processing techniques, such as filtering and picture open and close operations, will overwhelm the target light source, making accurate location difficult. Therefore, we introduce super-resolution generative adversarial networks (SRGAN) into the detection process and perform 4× upscaling on the identified beacon image. The feature information of underwater small targets can be effectively improved by using this technique, and it also offers a guarantee for the accuracy of subsequent subpixel centroid positioning based on Zernike moments.

The core of SRGAN consists of two networks: a super-resolution generator and a discriminator, where the discriminator uses the VGG19. First, apply a Gaussian filter to a real high-resolution image ${\hat{I}}^{\mathrm{HR}}$ with channel and size C×W×H to obtain a low-resolution image $I^{\mathrm{LR}}=C\times rW\times rH$, where the scaling factor is r.This low-resolution image is then used as input to the generator and trained to produce a high-resolution image $I^{\mathrm{HR}}$. The original high-resolution image ${\hat{I}}^{\mathrm{HR}}$ and the generated image $I^{\mathrm{HR}}$ are both input to the discriminator to obtain the perceptual loss function $l^{\mathrm{SR}}$ of the generated image and the real image, which includes the content loss $l_{VGG/i,j}^{SR}$ and the adversarial loss $l_{Gen}^{SR}$ [23]. Then the relationship between the losses can be obtained as

(4) $$l^{\mathrm{SR}}=l_{VGG/i,j}^{SR}+10^{-3}l_{Gen}^{SR},$$

where

$$l_{VGG/i,j}^{SR}=\frac{1}{W_{i,j}H_{i,j}}{\sum_{x=1}^{W_{i,j}}\sum_{y=1}^{H_{i,j}}\left(\phi_{i,j}{\left(I^{HR}\right)}_{x,y}-\phi_{i,j}{\left(G_{\theta G}\left(I^{LR}\right)\right)}_{x,y}\right)}^{2},$$

$$l_{Gen}^{SR}=\sum_{n=1}^{N}-\log D_{\theta D}\left(G_{\theta G}\left(I^{LR}\right)\right).$$

In Formula (4), $G_{\theta G}$ is the reconstructed high-resolution image, $D_{\theta D}$ is the probability that an image belongs to a real high-resolution image, i and j represent the ith maximum pooling layer and the jth convolution layer in the VGG network, respectively. We used the high-definition camera Cannon D60 to collect 7171 high-definition images of underwater optical beacons and trained 400 rounds under the condition of a scaling factor of 4. At the same time, in order to verify the advantages of SRGAN, we used the results generated by the SRResNet for comparison.

**Figure 13:**
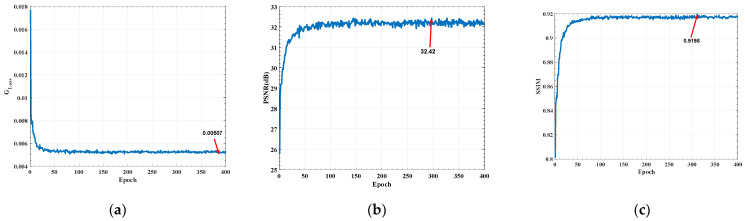
SRGAN training results: (**a**) Generator loss; (**b**) Peak signal-to-noise ratio; (**c**) Structural similarity index measurement.

Figure 13 shows the training result of SRGAN, where the generator loss (Figure 13a) drops to $5.07\times 10^{-3}$ in 400 epochs, indicating that the model converges well. Figure 12b is the peak signal-to-noise ratio (PSNR), which is mainly aimed at the error between the corresponding pixels of the generated image and the original image. The PSNR of the generated image can reach up to 32.42 dB. Structural similarity index measurement (SSIM) is an index used to measure the similarity of brightness, contrast, and structure between the generated image and the original high-resolution image. It can be seen that the maximum SSIM of the generated image can reach 91.98% (Figure 13c). The aforementioned data demonstrates that this method produces low levels of image distortion, and the image has good structural integrity and structural details.

**Figure 14:**
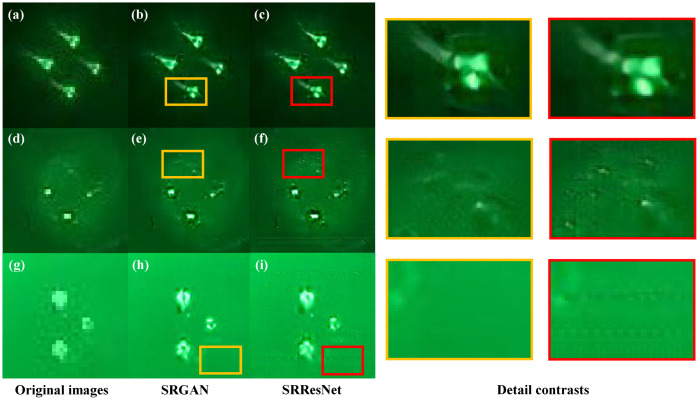
Comparison of the original images and the super-resolution reconstruction images: (**a**,**d**,**g**) are the original target images; (**b**,**e**,**h**) are super-resolution images of SRGAN; (**c**,**f**,**i**) are super-resolution images of SRResNet (the yellow box represents the SRGAN image detail, and the red box represents the SRResNet image detail).

Figure 14 displays the low-resolution target images, the target images produced by SRGAN, and the target images produced by SRResNet in order to illustrate the benefits of SRGAN. It can be clearly seen that the image generated by SRResNet will produce blurring problems at the edge of the target light source (Figure 14c); noise points will also appear near the target feature points (Figure 14f); the background will produce grid noise (Figure 14i). These noises all lead to errors in the process of light source centroid localization, while the images generated by SRGAN have good structural integrity and less noise (Figure 14b,e,h).

**Figure 15:**
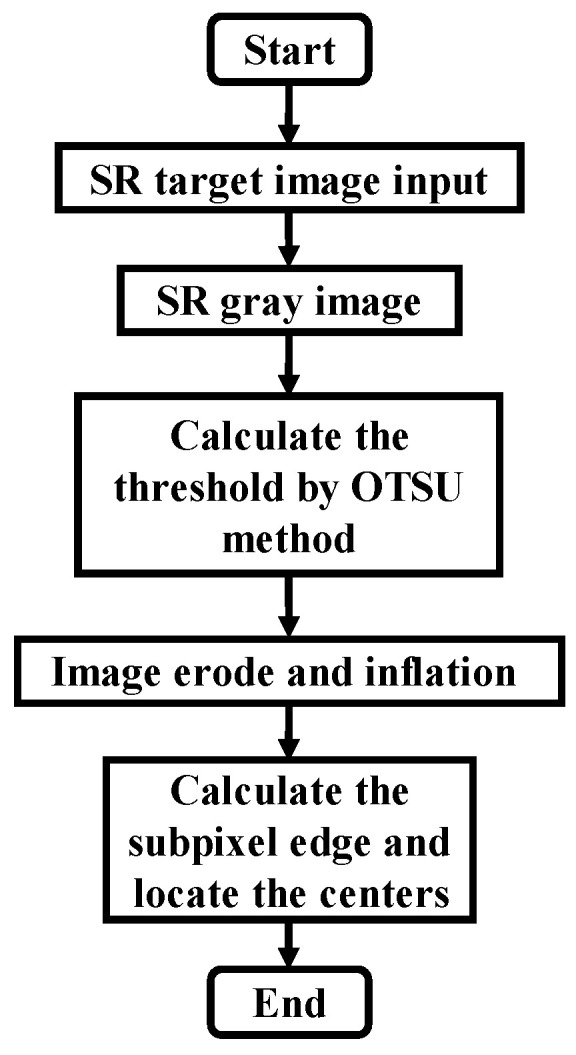
Sub-pixel optical center positioning flow chart.

Accurately locating the center of each light source in the image is a key step. The process of the centroid extraction stage is shown in Figure 15: Firstly, the OTSU method is selected for threshold segmentation, and then the image is corroded and expanded to smooth the light source edge to obtain the regular light source edge. The sub-pixel edge refinement method based on the Zernike moment is used to obtain the edge of the sub-pixel light source. Finally, the centroid coordinates of each light source are extracted by the centroid formula.

The difference between the background of the feature image and the target light source after YOLO V5_CA extraction and super-resolution enhancement has been obvious, so the OTSU method has been selected as the threshold segmentation algorithm. The OTSU method can effectively separate the target light source part and the background part from the foreground. It defines the segmentation threshold as a solution to maximize the inter-class variance. The scheme is established as follows:

(5) $$\sigma^{2}=\omega_{1}\cdot {\left(g_{1}-g_{e}\right)}^{2}+\omega_{2}\cdot {\left(g_{2}-g_{e}\right)}^{2},$$

where the between-class variance is denoted as $\sigma^{2}$, $\omega_{1}$ and $\omega_{2}$ are the probabilities that one pixel belongs to the target area or the background area, respectively. $g_{1}$ and $g_{2}$ are the average gray values of the target and the background pixels, respectively, while $g_{e}$ is the average gray value of all pixels in the image. Assuming that the image is segmented into the target area and the background area when the gray segmentation threshold is k, the first-order cumulative moment $g_{k}$ of k is brought into Formula (5):

(6) $$\sigma^{2}={\frac{\left(g_{e}\cdot \omega_{1}-g_{k}\right)}{\omega_{1}\cdot \left(1-\omega_{1}\right)}}^{2}.$$

By traversing the 0–255 gray values in Formula (6), the corresponding $\sigma^{2}$ is the required threshold when the variance between classes is the largest. There is a great difference between the light source characteristics and background characteristics of an underwater optical beacon, so the OTSU method is stable.

To precisely determine the pixel coordinates of each light center, it is necessary to obtain the edges of each light source after obtaining the binary image following threshold segmentation. Therefore, a sub-pixel edge detection method based on the Zernike moment is used. This method is not affected by image rotation and has good noise endurance. The n-order and m-order Zernike moments of the image f(x,y) are defined as follows:

(7) $$Z_{nm}=\frac{n+1}{\pi }\iint_{x^{2}+y^{2}\leq 1}f\left(x,y\right)V_{nm}^{*}\left(\rho ,\theta \right)dxdy,$$

where $V_{nm}^{*}\left(\rho ,\theta \right)$ is conjugate to the orthogonal n-order and m-order Zernike polynomial $V_{nm}\left(\rho ,\theta \right)$ of the polar coordinate system unit circle. Assuming the ideal edge rotates θ, because of the rotational invariance of Zernike moments: ${Z^{\prime }}_{00}=Z_{00}$, ${Z^{\prime }}_{11}=Z_{11}e^{i\theta }$, ${Z^{\prime }}_{20}=Z_{20}$, the sub-pixel edge of the image can be represented by Formula (8):

(8) $$\left[\begin{array}{c} x_{s}\\ y_{s} \end{array}\right]=\left[\begin{array}{c} x\\ y \end{array}\right]+\frac{Nd}{2}\left[\begin{array}{c} \cos\varphi \\ \sin\varphi \end{array}\right].$$

In Formula (8), d is the distance from the center of the unit circle to the ideal edge in the polar coordinate system, and N is a template for Zernike moments, which improves the accuracy as its size increases but increases the calculation time.

**Figure 16:**
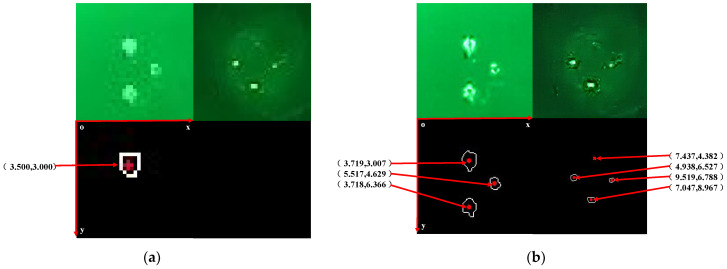
Comparison between the traditional algorithm and the subpixel centroid localization method based on SRGAN and Zernike moments (the top-row images are targets, and the bottom-row images are results): (**a**) Results of OTSU threshold segmentation + Zernike moment sub-pixel center search; (**b**) Results of our method.

Figure 16 shows the detection comparison of the low-resolution target images and the super-resolution enhanced images using the method described in this paper. It is evident that the direct OTSU and Zernike edge detection algorithms are unable to find all of the light source centers when the target light source structure is incomplete. When the light source is far away, the light source center cannot be detected due to the small number of pixels occupied by each light source. The super-resolution enhancement and Zernike moment sub-pixel detection method can well extract the target sub-pixel center coordinate accuracy of each light source to 0.001 pixels, which provides a guarantee for subsequent accurate attitude calculation.

## 4. Experiments on Algorithm Accuracy and Performance

Under the condition that the feature points in the world coordinate system of the object and its corresponding pixel coordinates in the image coordinate system are known, the problem of solving the relative position between the object and the camera is called the perspective-n-points (PnP) problem. Accurately solving this problem generally requires more than four known corresponding points. This section has done the following experiments:

1. Compare the traditional PnP algorithms, OPnP, LHM decomposition, and SRPnP in solving the coplanar 4-point small optical beacon translation distance error;
2. Compare the accuracy of the traditional algorithm with the method described in Section 3;
3. Compare the running speed of the algorithm before and after adding the super-resolution enhancement.

**Figure 17:**
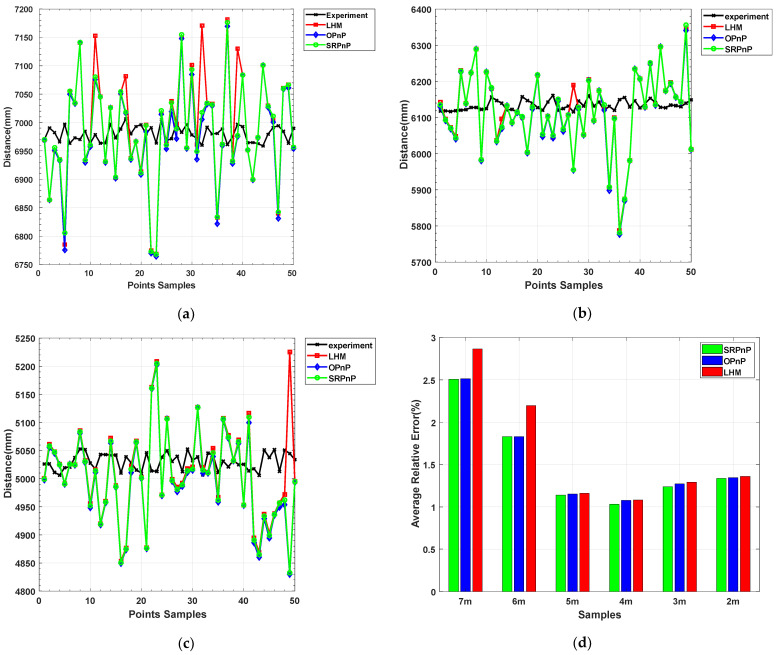
Traditional PnP algorithm distance detection results: (**a**) Detection results of optical beacons within 7 m; (**b**) Detection results of optical beacons within 6 m; (**c**) Detection results of optical beacons within 5 m; (**d**) The average relative error of translation.

In order to compare the average relative error and range accuracy of the OPnP, LHM, and SRPnP algorithms, 9 groups of 450 sample data were sampled 50 times every 1 m in the range of 10–2 m. The average relative errors of the three algorithms are shown in Figure 17. The average detection distance and experimental data of the three algorithms are shown in Table 1.

**Figure 18:**
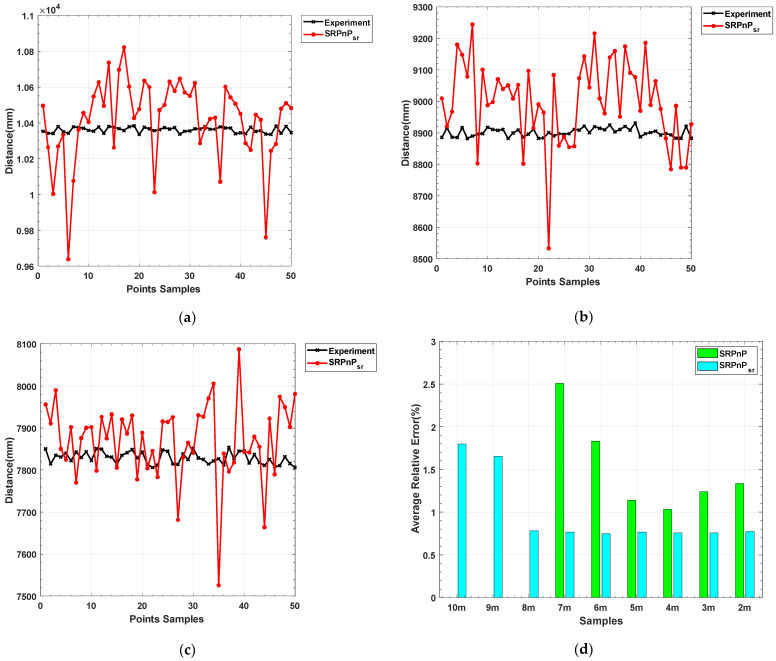
Ranging results are based on super-resolution image enhancement and a subpixel centroid localization method: (**a**) Detection results of optical beacons within 10 m; (**b**) Detection results of optical beacons within 9 m; (**c**) Detection results of optical beacons within 8 m; (**d**) The average relative error of translation.

The experimental data in Table 1 was measured using a high-precision translation platform. The 50 samples in the PnP algorithm solution data of each group are randomly obtained from the shooting videos at different distances. By analyzing the data, it can be concluded that when the small optical beacon is far away from the camera (10–7 m), the traditional PnP algorithm cannot be solved because it cannot obtain the coordinates of the four feature points in the pixel coordinate system. In the middle and long distance (5–7 m) range, the accuracy of the three LHM iterative algorithms is low, and the average relative error is about 2.53%. Overall, the solution accuracy of SRPnP and OPnP algorithms is not much different, and the average relative errors are 1.51% and 1.53%, respectively. In order to reflect the accuracy and the detection range of our method, it is compared with SRPnP. The results are shown in Figure 18.

By examining the data in Figure 18, it can be seen that the issue with the feature points being unable to be recognized and located at a great distance (10–7 m) has been resolved, and the feature point extraction range of the remote small optical beacon has been greatly improved. The average relative error of the SRPnP algorithm in solving a 10–7 m target is 1.25%, and the average relative error in short distances (within 7 m) is 0.83%. From the above data, we can see that our method reduces the calculation error by 33.6% and improves the calculation accuracy.

**Figure 19:**
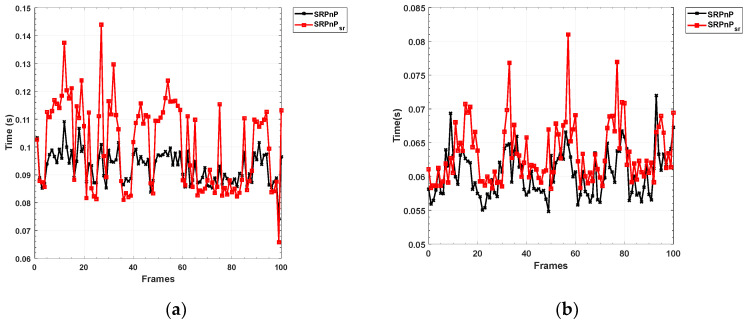
Comparison of detection speeds of each frame image (**a**) The operating speed of algorithm in 5 m; (**b**) The operating speed of algorithm in 10–5 m.

In order to further reflect the efficiency of the algorithm, our algorithm and the traditional algorithm are compared in time under the same hardware and software conditions. The video capture rate of the camera used in the experiment is 20 FPS, and the algorithms used in this experiment are tested on a personal computer equipped with an i7-8750 CPU, 32 g of memory, and Windows 10. We used Visual Studio 2017 and Qt 5.9.8 for the pose algorithm implementation and neural network migration without GPU acceleration. In Figure 19, the computing time of our method is 0.063 s (15.87 FPS) at a long distance, and the computing time of the SRPnP algorithm is 0.060 s (16.67 FPS). The computing time of our method is 0.101 s (10.17 FPS) at a short distance, and the com-puting time of the SRPnP algorithm is 0.093 s (10.83 FPS). The average detection speed of our algorithm in the whole range is 0.088 s (11.36 FPS). It can be seen that the time consumption of the two methods is not much different. This is because the size of the target image is small, and the combination of super-resolution and sub-pixel algorithm has a small impact.

**Figure 20:**
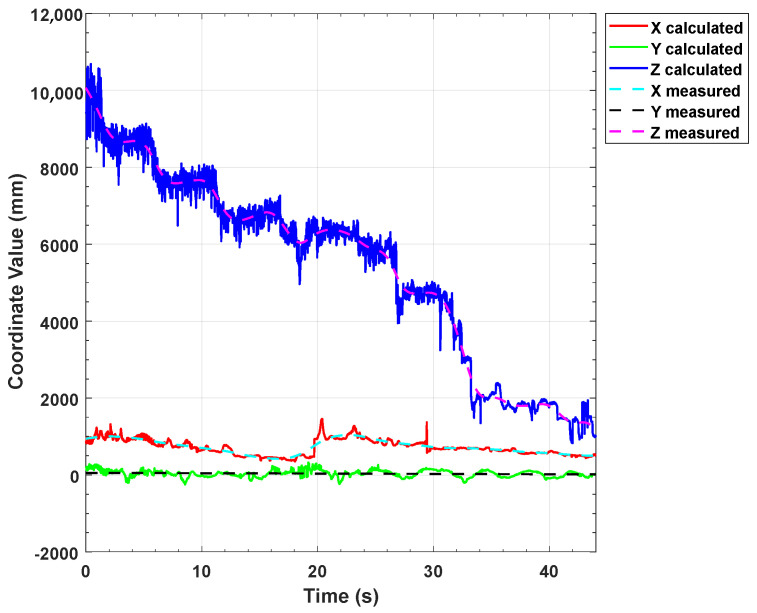
Dynamic positioning experiment results.

Figure 20 is a graph of the dynamic positioning results of our algorithm, in which the solid lines are the solution results in the X, Y, and Z axis directions, respectively, and the dotted lines are the readings of the moving platform. It can be seen from the results that our method has high detection accuracy and the advantage of real-time performance.

**Figure 21:**
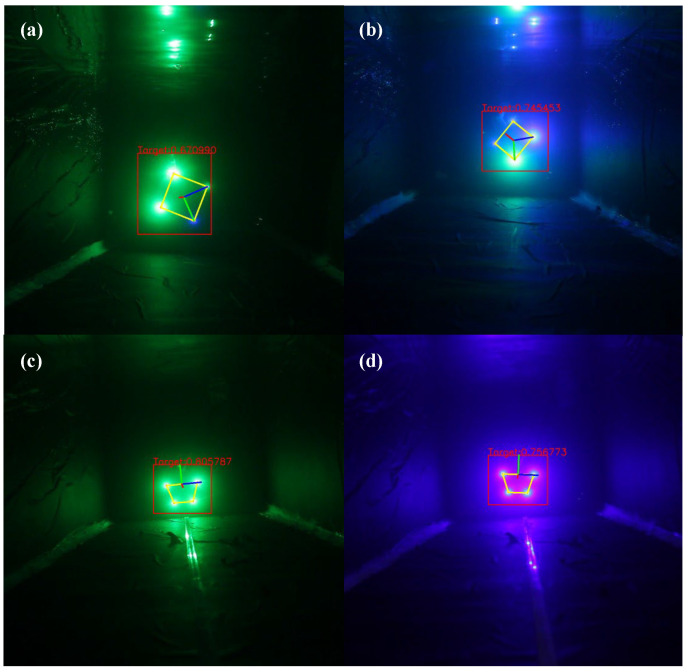
Underwater optical beacon attitude calculation effect diagram: (**a**) Optical beacon of the green cross; (**b**) Optical beacon of the blue cross; (**c**) Optical beacon of the green trapezoid; (**d**) Optical beacon of the blue trapezoid.

In this paper, LED light beacon arrays of different colors and shapes are designed to verify the accuracy of the algorithm, as shown in Figure 21. Therefore, if the ranging experiment of the system with multiple AUVs is designed, the color recognition function can be added after the target detection to perform ranging detection on the installation of different types of optical beacons. It can be seen that the method described in this paper has a good solution effect, and it also has a good discrimination effect on the illusion of water surface and lens glass.

## 5. Discussion

Table 2 shows the performance comparison between the existing underwater optical beacon detection algorithm and the algorithm described in this paper. It can be seen that the algorithm in this paper has high accuracy and a long detection range for optical beacons much smaller than the conventional size, and the algorithm’s time-consuming does not increase significantly. This shows that this paper provides an efficient and accurate optical beacon finding and positioning method for the end-docking of small AUVs.

## 6. Conclusions

In this paper, a quick underwater monocular camera positioning technique for compact 4-light beacons is presented. It combines deep learning and conventional image processing techniques. The second part introduces the experimental equipment and system in detail. A YOLO v5 target detection model with a coordinated attention mechanism is constructed and compared with the original model and the model with CBAM. The model has a classification accuracy of 96.1% for small optical beacons, which is 1.5% higher than the original network structure, and the recall is also increased by 0.8%. A sub-pixel centroid localization method combining SRGAN super-resolution image enhancement and Zernike moments is proposed, which improves the feature localization accuracy of small target light sources to 0.001 pixels. Finally, experimental verification shows that our method extends the detection range of small optical beacons to 10 m, controls the average relative error of distance detection at 1.04%, and has a detection speed of 0.088 ms (11.36 FPS).

Our method proposes a feasible monocular vision ranging scheme for small underwater optical beacons, which has the advantages of fast calculation speed and high precision. The combination of super-resolution enhancement and sub-pixel edge refinement is not limited to underwater optical beacon finding in AUV docking, and it can also be extended to other object detection fields. For example, satellite image remote sensing and small target detection tasks in medical images.

However, our method also has certain limitations. For example, the optical beacons and laser probes used in the experiment are only fixed on the high-precision rotary device, which is limited by the fixing method of the equipment and the moving mode of the rotary device, which cannot simulate the problems faced by the dynamic docking of AUVs in real situations. In this paper, optical beacons of various shapes and colors are designed to face the problem of visual positioning in the multi-AUV working system. However, limited by the manufacturing cost of the equipment, the multi-AUV docking experiment has not been carried out. Therefore, fixing the optical beacon and the laser probe in the full-size AUV for sea trials, verifying the performance of the algorithm under dynamic conditions, and designing the visual recognition of the multi-AUV system are the next research directions.

## Figures and Tables

**Table 1:** Experimental data and algorithm detection results.

Sample Groups	Average Experiment Results (mm)	Average LHM Results (mm)	Average OPnP Results (mm)	Average SRPnP Results (mm)
1	10,344.00	None	None	None
2	8892.00	None	None	None
3	7815.00	None	None	None
4	6968.00	7167.80	7143.45	7143.00
5	6122.00	6256.45	6234.07	6234.07
6	5015.00	5095.59	5072.82	5075.22
7	4082.00	4126.44	4126.27	4124.44
8	3012.00	3050.97	3050.45	3049.41
9	1987.00	2014.62	2014.34	2014.14

**Table 2:** Performance comparison between existing algorithms and our algorithm.

Method	Optical Beacon Size (mm)	Detection Range (m)	Detection Speed (s)	Average Relative Error
R. L.’s [15]	100	3.6	0.015	2.00%
S. L.’s [16]	2014	6.5	0.120	0.14%
R. R.’s [17]	280	8.0	0.059	5.00%
Z. Y.’s [32]	600	4.5	0.050	4.44%
Ours	88	10	0.088	1.04%

## Data Availability

The data presented in this study are available on request from the corresponding author.
